# Evolution and diversity of periplasmic proteins involved in copper homeostasis in gamma proteobacteria

**DOI:** 10.1186/1471-2180-12-249

**Published:** 2012-11-02

**Authors:** Georgina Hernández-Montes, José M Argüello, Brenda Valderrama

**Affiliations:** 1 Departamento de Medicina Molecular y Bioprocesos. Instituto de Biotecnología, Universidad Nacional Autónoma de México, Av. Universidad 2001 Col. Chamilpa, Cuernavaca, Mor, CP 62210, México; 2 Department of Chemistry and Biochemistry, Worcester Polytechnic Institute, Gateway Park, 60 Prescott St, Worcester, MA, 01605, USA

**Keywords:** Copper homeostasis, Gamma-proteobacteria, Evolution

## Abstract

**Background:**

Different systems contributing to copper homeostasis in bacteria have been described in recent years involving periplasmic and transport proteins that provide resistance via metal efflux to the extracellular media (CopA/Cue, Cus, Cut, and Pco). The participation of these proteins in the assembly of membrane, periplasmic and secreted cuproproteins has also been postulated. The integration and interrelation of these systems and their apparent redundancies are less clear since they have been studied in alternative systems. Based on the idea that cellular copper is not free but rather it is transferred via protein-protein interactions, we hypothesized that systems would coevolve and be constituted by set numbers of essential components.

**Results:**

By the use of a phylogenomic approach we identified the distribution of 14 proteins previously characterized as members of homeostasis systems in the genomes of 268 gamma proteobacteria. Only 3% of the genomes presented the complete systems and 5% of them, all intracellular parasites, lacked the 14 genes. Surprisingly, copper homeostatic pathways did not behave as evolutionary units with particular species assembling different combinations of basic functions. The most frequent functions, and probably because of its distribution the most vital, were copper extrusion from the cytoplasm to the periplasm performed by CopA and copper export from the cytoplasm to the extracellular space performed by CusC, which along with the remaining 12 proteins, assemble in nine different functional repertoires.

**Conclusions:**

These observations suggest complex evolutionary dynamics and still unexplored interactions to achieve copper homeostasis, challenging some of the molecular transport mechanism proposed for these systems.

## Background

Copper atoms in cuproenzymes alternate between oxidation states (II)/(I) with oxidation potentials ranging between + 0.25 and + 0.75 V [1] . The ability of cuproenzymes to exploit these high potentials and to perform redox reactions is widespread playing key roles in electron transfer and in oxygen transport and activation. However, high concentrations of intracellular copper are toxic for cells. Cu(I) has been shown *in vitro* to activate oxygen or hydrogen peroxide and to perform Fenton chemistry [2]. However, it has been reported that copper does not catalyze significant oxidative DNA damage *in vivo*; therefore, copper toxicity must occur by a different mechanism, possibly by disruption of FeS clusters [3]. In response to its toxicity, cells keep copper concentration under strict control allowing enough metal to be available for protein assembly but below damage induction threshold [4].

**Figure 1:**
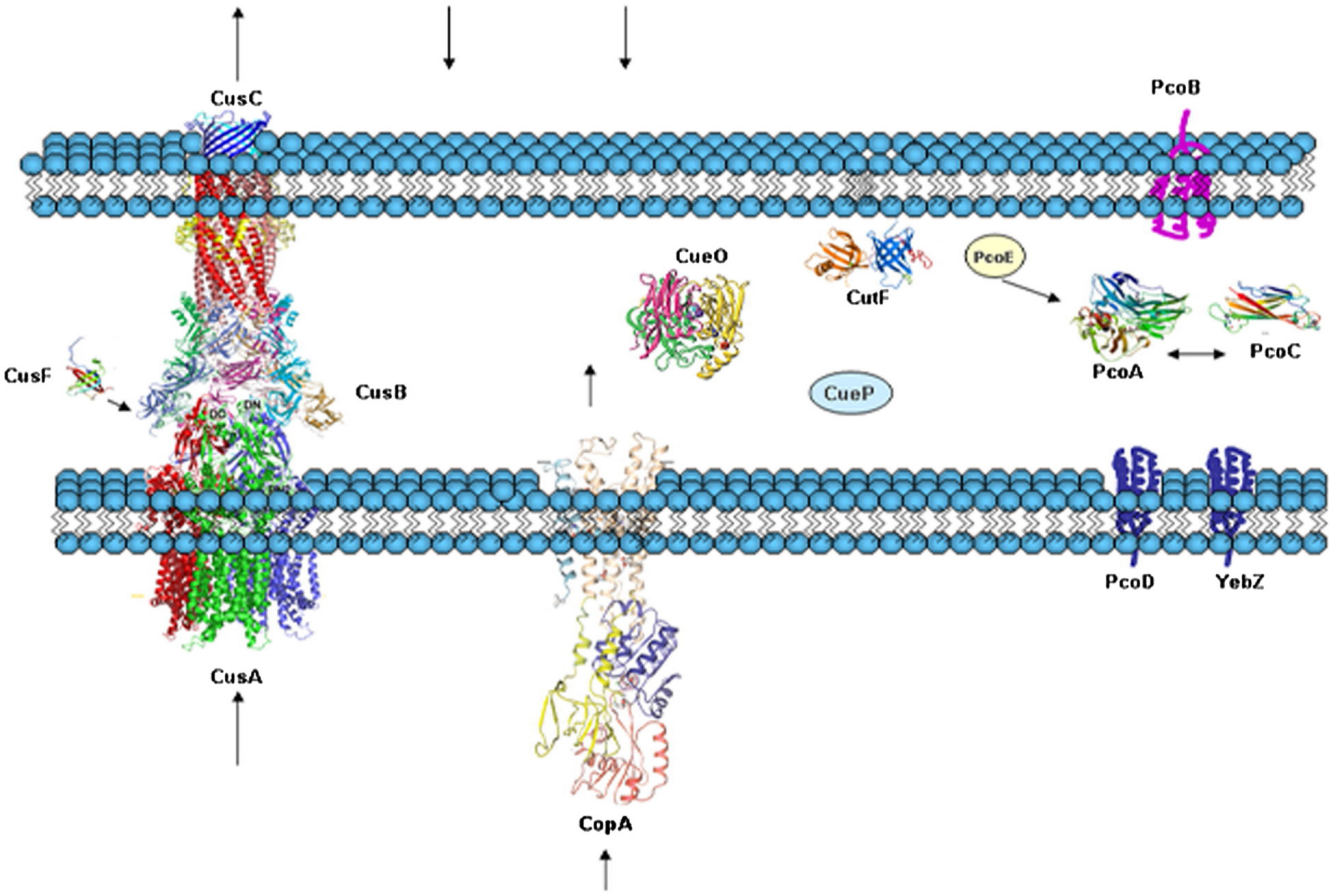
**Graphical view of the complete copper homeostasis ensemble in gamma proteobacteria depicting the five systems identified to date: CopA, CueO and CueP belong to the Cue system; CusA, CusB, CusC and CusF belong to the Cus system; PcoA, PcoB, PcoC, PcoD and PcoE belong to the Pco system; CutF belongs to Cut system and YebZ, not yet ascribed to a system, is a PcoD homolog.** Arrows indicate copper ions described currents.

Current knowledge of copper homeostasis systems in bacteria has been elucidated from the study of gamma proteobacteria such as *Salmonella enterica* sv. Typhimurium [5]*, Shigella flexneri*[6] and *Escherichia coli*[7]. In these organisms, the archetypical copper resistance response involves the coordinated function of four different systems: CopA/Cue, Cus, Pco and Cut, responsible for copper import, export or detoxification. A set of copper-sensing transcriptional regulators (CueR, CusR, CusS, PcoR and PcoS) specifically modulate the expression of these genes [8]. For instance, in *E. coli* under aerobic conditions, CueR activates the expression of *copA* and *cueO*, encoding for a periplasmic multi-copper oxidase (MCO). CueR also induces expression of *cueP*, encoding for a periplasmic protein of unknown function putatively involved in copper-resistance in *Salmonella*[5]. While CopA pumps out excess copper from the cytoplasm to the periplasm, CueO oxidizes Cu(I) to Cu(II) in periplasm thereby reducing Cu(I) concentration [9,10]. Under anaerobic conditions, CusR and CusS activate the transcription of the *cusCBAF* operon that encodes for a complex that pumps Cu(I) to the extracellular space [11]. This complex consists of the inner membrane pump CusA, the periplasmic protein CusB and the outer membrane protein CusC forming a channel through the periplasm. CusF has been proposed to feed the CusABC channel with copper from the periplasmic space [12]. PcoR and PcoS are transcriptional regulators for the copper-inducible expression of the *pcoABCD* operon [13]. *pcoA* encodes for a periplasmic MCO. There is no known function for PcoB although it may function as an outer membrane protein. PcoC is a periplasmic copper carrier with two metal binding sites selective for Cu(I) or Cu(II) and has been suggested to interact with PcoD (an integral membrane protein) in copper translocation into the cytoplasm. *pcoE* apparently encodes for a cytoplasmic protein with a putative function as a copper scavenger. There is no information available regarding the regulation of the Cut system that involves at least six proteins: CutA, CutB, CutC, CutD, CutE, and CutF [14]. CutF and CutC have been described as involved in copper tolerance in *E.coli*. Since CutC is a cytoplasmic protein perhaps involved in intracellular trafficking of Cu(I), while CutF is an outer membrane protein [15], we only included CutF in our analysis Figure 1.

The process by which bacteria handle copper can be seen in a manner analogous to a metabolic pathway since organisms avoid free copper ions within the cell by developing copper translocation routes based in precise sequences of specific protein-protein interactions [16-18]. Evolution of these pathways should be hence reflected in the correlative evolution of interacting partners. Based on this idea, we hypothesized that traffic/transport systems would be constituted by a defined set of essential components, probably related by co-regulation, and thus to co-evolve. We have analyzed the distribution in gamma proteobacteria of all proteins known to be involved in copper homeostasis to identify the minimal sets of elements involved in copper homeostasis and to propose an evolutionary model.

## Results

### Orthologs identification and profile construction

We selected 14 different proteins known to be involved in copper homeostasis from three gamma proteobacterial isolates as seeds for BLAST searches of their orthologs: five proteins from *Escherichia coli* K12 MG1655 (CopA, CusA, CusB, CusC and CusF), eight proteins from *Escherichia coli* O1:K1:H7 (APEC) (PcoA, PcoB, PcoC, PcoD, PcoE, CueO, YebZ and CutF), and one protein from *Salmonella enterica* subsp. enterica serovar Typhimurium LT2 (CueP).

Ortholog assignment was performed using the Bidirectional Best Hit (BBH) criterion. The best hit of a seed sequence in a target genome is the gene in that genome that represents the best match. The best hit is bidirectional if both sequences (seed and target) result to be the best hit for each other [19].

**Figure 2:**
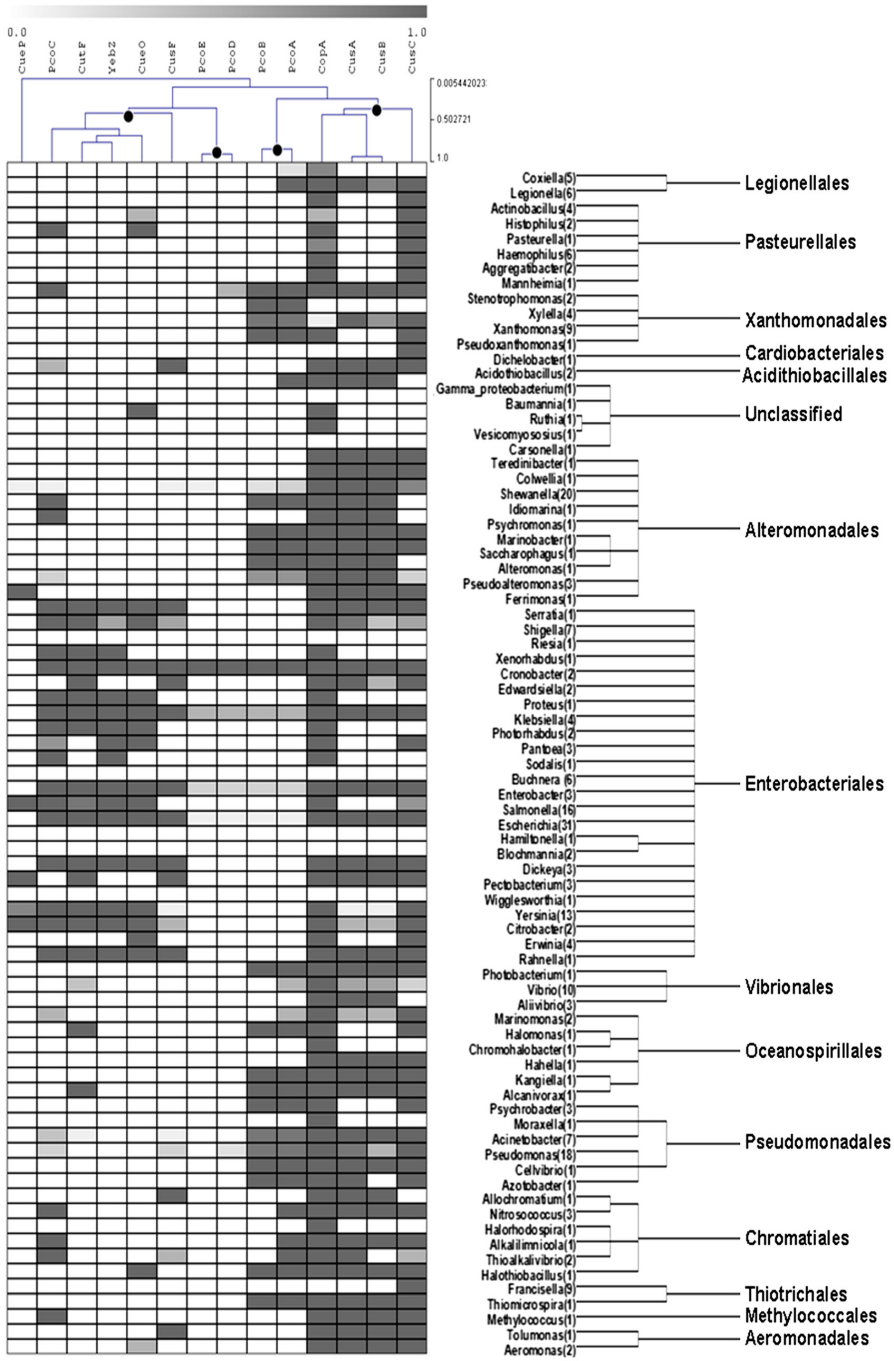
**Hierarchical clustering of the taxonomical distribution of periplasmic copper homeostasis proteins.** Optimization of the profile by protein presence with clades representing taxonomic categories at the genus level organized according to their phylogenetic distribution. Five protein clusters were identified (marked with dots) according to their clustering value as described in Materials and Methods. Shade scale represents the fractional abundance of a seed protein within a genus, a value corresponding to the percentage of genomes where a given ortholog was identified. The number of genomes in each genus is indicated in parenthesis.

Analysis of 268 gamma proteobacterial genomes (Additional file 1) by BBH criterion allowed the identification of 1,417 orthologs to the seed proteins. The abundance of the proteins in the ensemble was 85% for CopA, 77% for CusC, 60% for CusA, 53% for CusB, 42% for PcoC, 37% for CueO, 36% for CutF, 33% for YebZ and PcoA, 26% for CusF, 25% for PcoB, 13% for CueP, and 4% for PcoD and PcoE. This information was transformed into a presence/absence matrix by assigning a presence value of one when an ortholog was identified in a genome and a value of zero when not. In order to eliminate the redundancy derived from the over representation of certain species and to develop a better representation, information was consolidated at the genus level and organized in 11 discrete intervals between 0 (absence of an ortholog within a genus) and 1 (presence of an ortholog in 100% of the genomes within a genus). This value represents the fractional abundance of a seed protein within a genus (Figure 2). The distribution of the resultant 79 genera was fixed by their phylogenetic relationships and then the matrix subjected to a subordinated hierarchical clustering.

**Figure 3:**
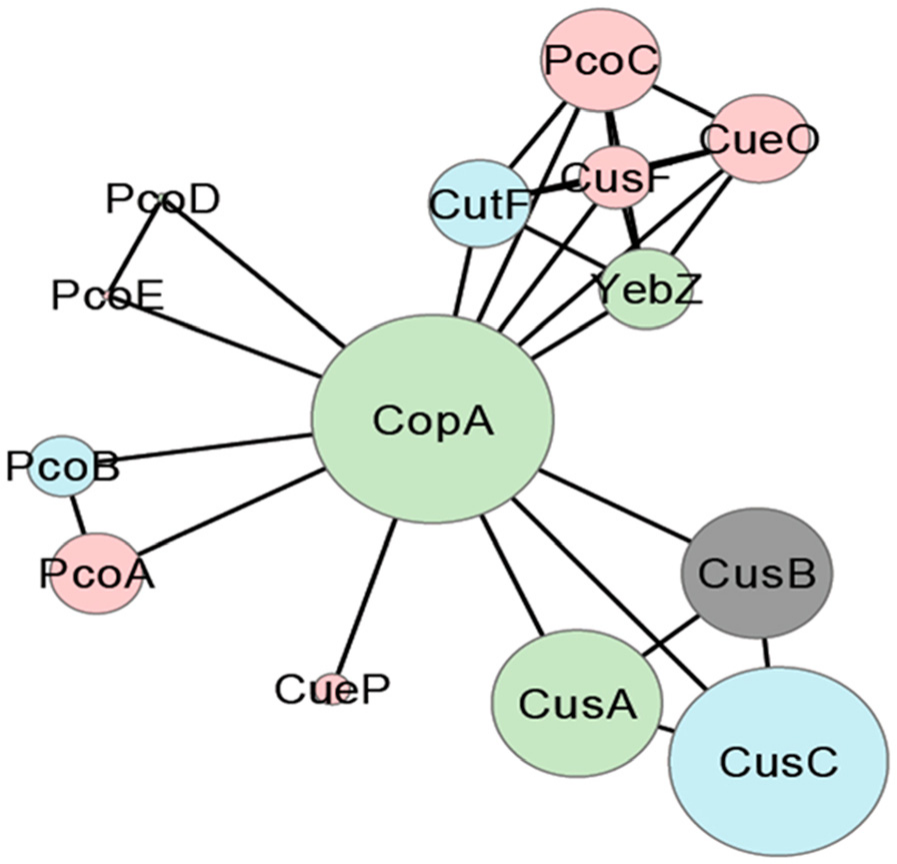
**Graphical representation of the complete periplasmic copper homeostasis ensemble in gamma proteobacteria.** Each circle represents a seed protein with circle size indicating its relative abundance in the ensemble (CopA circle represents 100%). Proteins are distributed in five groups following the clustering analysis described in Figure 2. Lines indicate elements association within and between clusters (the length of the lines is not informative). Color key: Inner membrane proteins in green, external membrane proteins in blue, periplasmic soluble proteins in red, and CusB in grey.

It has been previously accepted that a Pearson coefficient between 0.75 and 0.9 is confident for data correlation assignment [20-22]. All the proteins in the ensemble, with the exception of CueP, distributed in four pairs below the correlation threshold value of 0.75: CusA-CusB, PcoE-PcoD, PcoA-PcoB, and YebZ-CutF with values of 0.92, 0.90, 0.83 and 0.77, respectively. With the exception of CueP, these pairs were further assembled with the rest of the proteins in four clusters keeping the affinity level over 0.5 as recommended [23,24]: PcoC-CueO-YebZ-CutF-CusF, PcoE-PcoD, PcoA-PcoB, CusC-CusA-CusB-CopA. In order to depict the relationships identified in Figure 2, we employed a graphical representation of the whole ensemble as a network with the most abundant protein (CopA) as the central node and the rest of the proteins distributed in accordance to the five defined clusters (Figure 3). The functional composition and genomic linkage of all the protein elements involved in the most frequent representation of each one of these clusters is presented in this section.

### PcoC-CutF-YebZ-CueO-CusF

This cluster comprises proteins from five different systems in two versions, with or without CusF, being the tightest pair in the cluster YebZ-CutF. YebZ is a homolog of PcoD and has been predicted to be an inner membrane protein whereas CutF belongs to the NlpE family and has been proposed to be an outer membrane protein. Both genes are relatively well represented in the ensemble with *yebZ* located in the genome of 88 Enterobacteria and *cutF* in the genome of 97 organisms from which 91% are Enterobacteria and the rest *Vibrio* (4%), *Pasteurella*, *Acinetobacter*, *Alcanivorax and Halomonas* (1% each). The stringent presence correlation of YebZ-CutF in 81 genomes of Enterobacteria cannot be explained by genetic linkage since in no case their genes are contiguous, suggesting strong functional compromise. Furthermore, *cutF* is not linked to any other gene encoding for copper homeostasis proteins.

CueO is a periplasmic MCO with activity of cuprous oxidase, *cueO* was located in the genome of 97 organisms from which 98% are Enterobacteria and the rest *Aeromonas* and *Halothiobacillus* (1% each). The genomic location of *cueO* is chromosomal in all analyzed organism and only in *Halothiobacillus neapolitanus* C2 it was found to be linked to other genes encoding for copper homeostasis proteins (*cusABC-cueO-pcoAB*). The presence of CueO with YebZ-CutF correlated in 78 genomes of Enterobacteria. In few cases such as in the genomes of four *Erwinia* species, in *Aeromonas hydrophila* subsp. hydrophila ATCC 7966 and in *Ruthia maifica* str. Cm, CueO was identified in the absence of the rest of the cluster.

The fourth element of the cluster is PcoC, a periplasmic copper carrier that has been proposed to interact with PcoA. The genomic location of *pcoC* is chromosomal with five exceptions (*Cronobacter turicensis* TAX413502, *Enterobacter cloacae* subsp. cloacae ATCC 13047, *Escherichia coli* APEC O1, *Klebsiella pneumoniae* subsp. pneumoniae MGH 78578 and *Klebsiella pneumoniae* NTUH-K2044). It is important to notice that these five organisms harbor the full copper homeostasis protein repertoire. PcoC was identified in the genomes of 110 organisms from which 81% were Enterobacteria and the rest Pseudomonadales (7%), Chromatiales (4%), Alteromonadales (3%), *Stenotrophomonas* (2%), *Acidiothiobacillus* and *Methylococcus* (1% each). Chromosomal copies of *pcoC* are contiguous to other genes encoding for copper homeostasis proteins in 85 cases as well as in five out of six plasmidic copies. The whole *pcoABCDE* system was identified in one *Cronobacter* and in two *Escherichia* chromosomes and in one *Cronobacter*, one *Escherichia* and two *Klebsiella* plasmids. Incomplete operons were also identified: *pcoABC* in *Shewanella*, *Idiomarina* and in one *Psudoalteromonas* plasmid and *pcoABCD* in three *Pseudomonas* chromosomes. A particular configuration was observed in *Enterobacter* where *pcoBCD* are contiguous in chromosome but *pcoAD* are plasmid borne. *pcoA* and *pcoC* coexist in 26 genomes from which 34% are Enterobactriales, 26% Alteromonadales, 19% Chromatiales, and 11% each Pseudomonadales and Xanthomonadales. In spite of its putative role as interacting partners *pcoA* and *pcoC* are contiguous in only 9 cases, four in chromosome and five in plasmids; however, in 87% of the genomes where they coexist, the chromosomal copies of *pcoC* are contiguous to *yebZ* and *yebY* but not to other members of the Pco system with the exception of the eight organisms with high protein number where *pcoC* is contiguous to *pcoD* (*Cronobacter turicensis* TAX413502*, Cronobacter sakazakii* ATCC BAA-894, *Enterobacter cloacae* subsp. cloacae ATCC 13047, *Klebsiella pneumoniae* subsp. pneumoniae MGH 78578, *Klebsiella pneumoniae* NTUH-K204 and *Escherichia coli 55989, ATCC 8739 and APEC0)*.

CusF was the fifth and the weakest element of this cluster. *cusF* was located in the genome of 70 organisms where 80% are Enterobacteriales and the rest Pseudomonales (10%), Alteromonadales (4%), Chromatiales and *Acidithiobacillus* (2% each). With a single exception, *Cronobacter turicensis* TAX413502, *cusF* was located in the chromosome. The functional role assigned to CusF is as a copper provider for the CusABC extrusion pump (located in a different cluster) however in only 62% of the cases their genes are contiguous and, in a single organism (*Thioalkalivibrio* sp. HL-EbGR7), *cusF* is contigous to *pcoA*.

### PcoE-PcoD

This cluster was exclusively found in organisms with large number of copper transport proteins*.* PcoD is a putative internal membrane protein and PcoE a copper chaperone. With the exception of *Enterobacter cloacae* subsp. cloacae ATCC 13047, *pcoE* and *pcoD* are contiguous with *pcoABC.* Particular arrangements were identified in two different *Enterobacter* species; in one *pcoE* and *pcoD* were located in the same plasmid although not contiguous and in the other one *pcoD* was plasmidic and *pcoE* chromosomal*.*

### PcoB-PcoA

This cluster was present in the genome of 67 organisms where 40% were Pseudomonales and the rest Xanthomonadales (22%), Altermonadales (15%), Enterobacteriales (12%), Oceanospirillales (6%), Chromatiales, Vibrionales and Thiotrichales (1.5% each). In 19 genomes *pcoA* was identified in the absence of *pcoB* but in no case was the opposite detected. *pcoA* and *pcoB* were contiguous in the chromosome of 82% of the organisms, contiguous in plasmids in 7.5% of the cases (*Cronobacter turicensis* TAX413502, *Escherichia coli* APEC O1, *Klebsiella pneumoniae* subsp. pneumoniae MGH 78578 and NTUH-K2044 and *Pseudoalteromonas haloplanktis* TAC125) and in a single case *pcoA* is plasmidic and *pcoB* chromosomal (*Enterobacter cloacae* subsp. cloacae ATCC 13047). In the genome of *Cronobacter turicensis* TAX413502 *pcoA* and *pcoB* were separated by a second copy of *pcoA*. In four genomes (*Enterobacter cloacae* subsp. cloacae ATCC 13047, *Pseudomonas putida* W619 and *Acinetobacter baumannii* SDF and AYE) the *pcoA* and *pcoB* identified orthologs belonged to two different *pcoAB* chromosomal operons.

### CopA-CusA-CusB-CusC

This cluster comprised three of the four members of the Cus system and CopA and was present in 119 organisms belonging to 21 families from 12 different orders (Acidithiobacillaes, Aeromonadales, Alteromonadales, Cromathiales, Enterobacteriales, Legionellales, Methylococcales, Oceanospirillales, Pseudomonadales, Thiotricales, Vibrionales and Xanthomonadales). The tightest pair was CusA-CusB, being CusA an internal membrane protein and CusB a periplasmic protein with the proposed role of connecting CusA and CusC. The presence of *cusA* and *cusB* correlated in 128 genomes belonging to 23 families from the same orders as listed above. In 92% of the cases where *cusA* and *cusB* coexist, they are contiguous in the chromosome or in plasmids. In five cases the *cusA* and *cusB* identified orthologs belonged to two different *cusAB* chromosomal operons (*Acidithiobacillus ferrooxidans*, *Legionella pneumophila* str. Lens*, Pseudomonas fluorescens* SBW25*, Saccharophagus degradans* Feb-40 and *Xanthomonas campestris* pv. vesicatoria str. 85*–*1). CusC was the second most abundant protein of the ensemble and its presence clearly correlated with CusA and CusB (124 out of 206 genomes); however the three genes are contiguous in only 44 Enterobacterial genomes. CopA, the most abundant protein of the sample with a physiological role as an internal membrane ATPase, was identified in the chromosomes of 70 genera with few exceptions: *Baumania, Buchnera, Coxiella, Dichelobacter,* one *Escherichia, Francisella,* two *Haemophilus, Wigglesworthia,* seven *Xanthomonas and Xylella*.

### CueP

CueP was found in 35 organisms from 6 genera (*Citrobacter, Salmonella, Pectobacterium, Yersinia, Ferrimonas* and *Shewanella*) belonging to only three families (Enterobacteriaceae, Ferrimonadaceae and Shewanellaceae). The presence correlation of CueP was the lowest of the experiment, coexisting with PcoC-CutF-YebZ-CueO and CopA-CusC in *Enterobacteriaceae* (ten *Yersinia*, one *Citrobacter* and sixteen *Salmonella*); with PcoC-CueO-YebZ-CutF, CopA-CusA-CusB-CusC and CusF in one *Yersinia* and one *Citrobacter*; with CopA-CusA-CusB-CusC and CusF or CutF in *Ferrimonas* and *Pectobacterium*; and with PcoA-PcoB, PcoC, PcoE, CopA-CusA-CusB-CusC and CusF in *Shewanella*.

From this analysis, an apparent phylogenetic consistency in the distribution of the clusters at the family level was evident.

### Double optimization and repertoire identification

**Figure 4:**
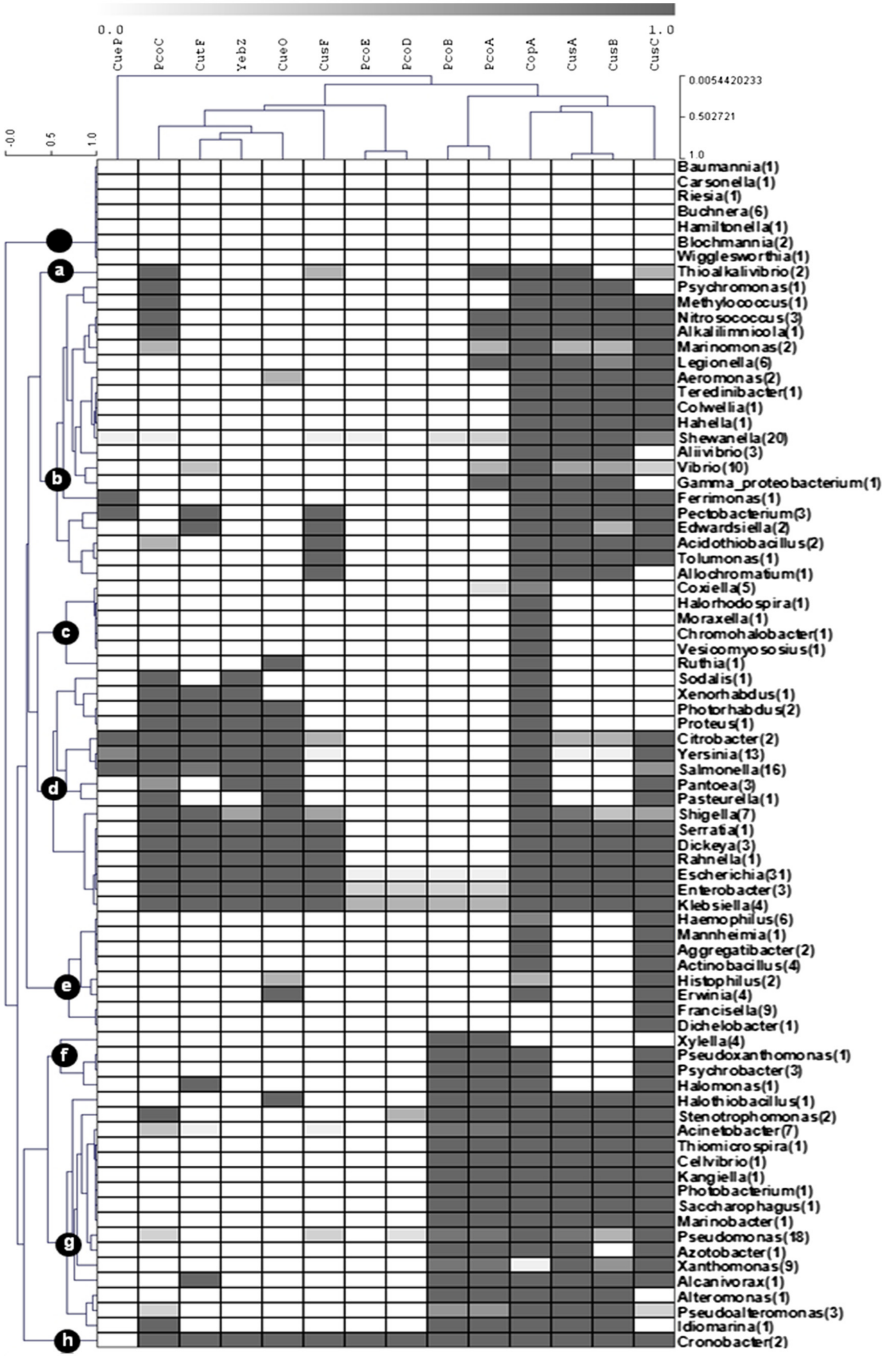
**Two-dimensional optimization of the phylogenetic profile of periplasmic copper homeostasis proteins.** Clustering optimization was rearranged for taxonomic categories preserving the previously optimized arrangement of protein presence. Eight proteins repertoires were identified (marked with dots). Shade scale represents the fractional abundance of a seed protein within a genus.

With the aim to identify particular combinations of the 14 seed proteins without the restrain imposed by a phylogenetic classification, we decided to perform the double optimization of the presence/absence profile (Figure 4). This analysis allowed the identification of nine clearly defined clades which represent the existing repertoires of periplasmic copper homeostasis proteins in gamma proteobacteria. In the first one (clade 0) we identified 13 organisms from seven genera that lack all seed proteins: *Baumannia, Carseonella, Riesia, Buchnera, Hamiltonella, Blochmannia* and *Wigglesworthia*. All these organisms are endosymbionts with reduced genomes suggesting the loss of copper homeostasis genes in response to the negligible role of copper homeostasis in their biological functions and environment.

**Figure 5:**
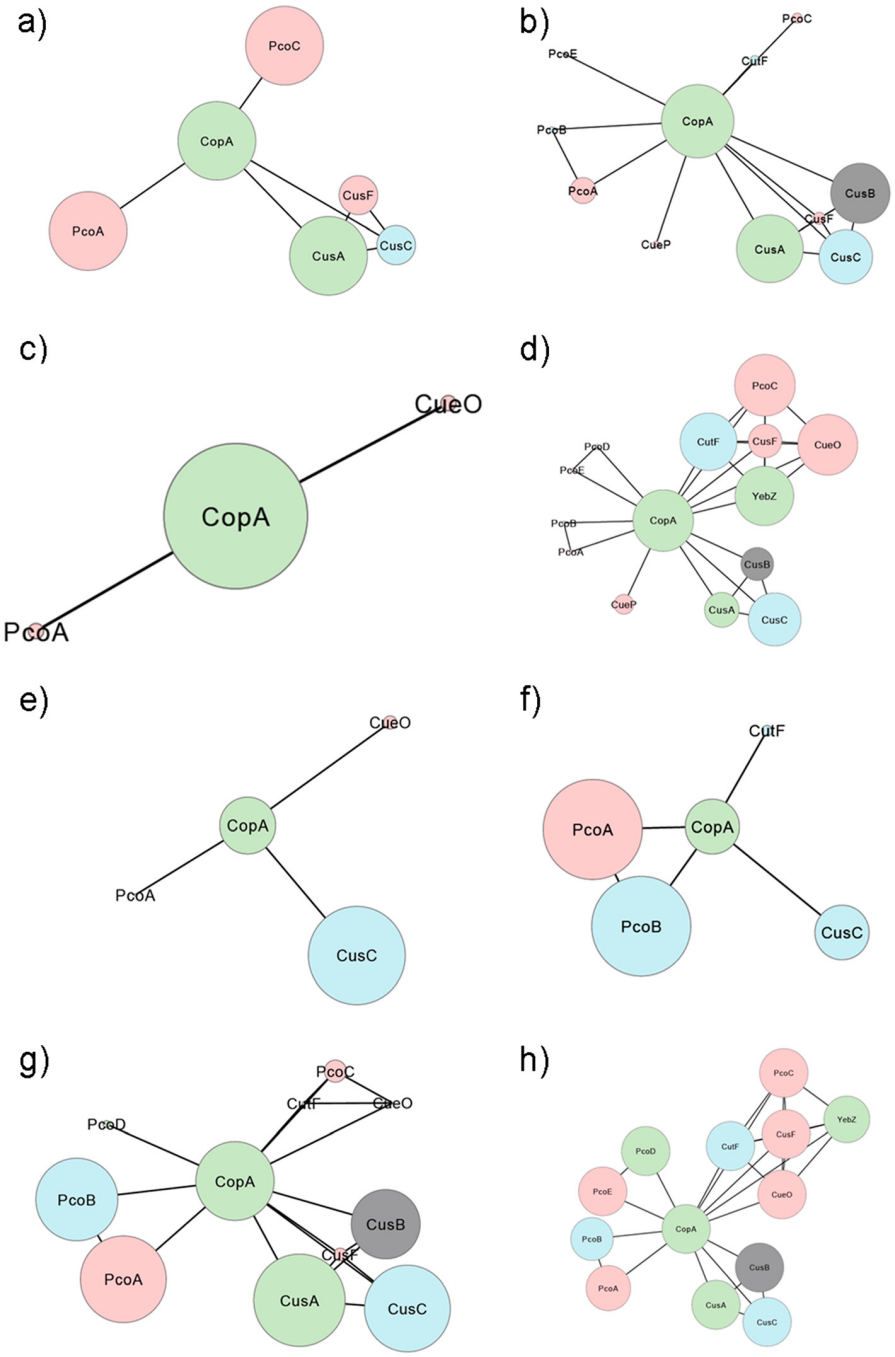
**Graphical representation of the different copper homeostasis repertoires identified in gamma proteobacteria by the two-dimensional optimization of the phylogenetic profile.** Each circle represents a seed protein and circle size its relative abundance within a repertoire. The size of the circle of the most abundant protein represents 100%. Color key: Inner membrane proteins in green, external membrane proteins in blue, periplasmic soluble proteins in red, and CusB in grey.

The second repertoire (clade 1) is depicted in Figure 5a and comprises two organisms from the same genus, *Thioalkalovibrio.* The core of this repertoire is formed by two inner membrane exporters (CopA and CusA), a pair of interacting periplasmic soluble carriers (PcoA and PcoC) and single elements of incomplete Cus systems (CusC or CusF). These organisms are highly haloalkaliphilic sulfur-oxidizing chemolithoautotrophs.

The third repertoire (clade 2) is depicted in Figure 5b and comprises 63 organisms from 15 families of 10 different orders. In this clade the core is formed by CopA and a partial Cus system (CusABC). Exceptions lacking CusA and/or CusB are *Marinomonas* sp. MWYL1 and 4 species of *Vibrio* and lacking CusC are *Psychromonas ingrahamii* 37, *Aliivibrio salmonicida* LFI1238, *Allochromatium vinosum* DSM 180 and *Gamma proteobacterium*. In the remaining organisms the core is accompanied by periplasmic carriers: CusF in *Pectobacterium*, *Edwardsiella*, *Acidithiobaciullus*, *Tolumona* and *Allochromatium*; CueP in *Ferrimonas* and *Pectobacterium*; PcoA and/or PcoC in *Psychromonas*, *Methylococcus*, *Nitrosococcus*, *Alkalilimnicola*, *Legionella*, *Shewanella*, *Vibrio* and *Acidithiobacillus*; and CueO in *Aeromonas*. CutF, an external membrane protein, was identified only in 4 species of *Vibrio*, *Ferrimonas* and *Pectobacterium*.

The fourth repertoire (clade 3) is depicted in Figure 5c and comprises 10 organisms from 6 genera, each one of a different family. This group contains only CopA as core protein and only 2 species an MCO (CueO in *Ruthia maifica* and *Coxiella burnetii* Dugway 5J108-111). The lifestyle of these organisms is diverse: two genera comprised halophilic free-living isolates (*Halorhodospora* and *Chromohalobacter*), two other genera comprised human pathogens (*Coxiella* and *Moraxella*) and the last two genera comprised clam symbionts (*Ruthia* and *Vesycomiosocius*). This wide versatility suggests thriving in soft environments that allow survival with the minimal function of copper active export from the cytoplasm to periplasm.

The fifth repertoire (clade 4) is depicted in Figure 5d and comprises 90 organisms from a single family (Enterobacteriaceae). This group contains the 14 seed proteins being the core formed by CopA and the PcoC-CutF-YebZ-CueO-CusF cluster, complete in 8 genera and incomplete in other 8. The second most frequent cluster was CusABC, complete in 8 genera, partial in 6 more and totally absent in the last 4. The Pco system was identified in only 8 species belonging to 3 genera: *Klebsiella, Escherichia* and *Enterobacter*. Finally, CueP was identified only in *Citrobacter, Yersinia* and *Salmonella*. Some of these isolates have been characterized as animal pathogens, however many of them belong to the normal gut flora.

The sixth repertoire (clade 5) is depicted in Figure 5e and comprises 27 organism from 8 genera where 6 belonged to the *Pasteurellaceae* family and the others to *Franciscella* and *Dichelobacter*. The core of this repertoire is CusC and CopA with the exception of *Franciscella*, *Dichelobacter nodosus* VCS1703A and *Haemophilus somnus* 129PT lacking the last protein. Two genera contain a periplasmic carrier, CueO in *Erwinia* and PcoA in *Francisella philomiragia* subsp. philomiragia ATCC 25017. With few exceptions, the organisms in this clade are human, animal or plant pathogens.

The seventh repertoire (clade 6) is depicted in Figure 5f and comprises four *Xylella fastidiosa* isolates, three *Psychrobacter* species, *Halomonas elongata HELO_1864* and *Pseudoxanthomonas suwonensis.* The core of this repertoire is PcoA and PcoB as identified in *Xylela fasitidiosa*, a plant pathogen. Secondary elements were CopA and CusC, identified in the three *Psychrobacter* species, in *Pseudoxanthomonas suwonensis* and in *Halomonas elongate.* The latter organism also presented CutF. *Psychrobacter* and *Halomonas* are halophilic bacteria whereas *Pseudoxanthomonas* is a BTEX (benzene, toluene, ethylbenzene, and o-, m-, and p-xylene) degrader.

The eighth repertoire (clade 7) is depicted in Figure 5g and comprises 50 organisms from 16 genera of 9 families: Pseudomonadaceae, Halothiobacillaceae, Idiomarinaceae, Alcanivoracaceae, Alteromonadaceae, Moraxellaceae, Piscirickettsiaceae, Vibrionaceae and Xanthomonadaceae. The core of this repertoire is formed by CopA, CusABC and PcoAB which is shared by 10 genera. Exceptions are *Alteromonas macleodii*, *Idiomarina loihiensis* L2TR and two species of *Pseudoalteromonas* (lacking CusC); *Azotobacter vinelandii* and nine species of *Pseudomonas* (lacking CusB) and eight species of *Xanthomonas* (lacking CopA). Periplasmic carriers were identified as secondary elements: CueO in *Halothiobacillus neapolitanus*; CusF in five *Pseudomonas* species and *Acinetobacter baumannii* ATCC 17978; and PcoC in five *Pseudomonas* species (not the ones with CusF) and three *Acinetobacter* species (including *baumannii*). This is a highly diverse group of free-living species of soil and marine environments. This clade along with clade F comprises all the organisms belonging to orders Pseudomonadales and Xanthomonadales.

The ninth and last repertoire (clade 8) comprises two species form a single genus, *Cronobacter,* and is depicted in Figure 5h. In these species the repertoire is the largest, lacking only CueP, and equivalent to the one identified in other Enterobacterial species such as *Klebsiella*, *Enterobacter* and *Escherichia*. *Cronobacter* species are found in natural environments such as water, sewage, soil and vegetables. They are not usually enteric pathogens, although they can get to be opportunistic pathogens infecting and persisting in human macrophages. Apparently these organisms have a large number of virulence factors but there is no direct indication to the necessity for such a complete copper homeostasis repertoire.

## Discussion

We hypothesized that a more complete representation of the periplasmic proteins involved in copper homeostasis might provide insight in the mechanism of copper traffic and transport, apparent redundancies, key singular roles and evolutionary processes. Our strategy based in the identification of orthologs of 14 seed proteins involved in copper homeostasis in 268 gamma proteobacterial genomes from 79 genera. This data was further transformed into a presence/absence matrix and optimized, preserving the phylogenetic relationships of the organisms.

It was striking to observe that only 3% of the organisms present the full copper homeostasis proteins repertoire that was previously described in *E.coli*[7]. Interestingly, isolates presenting a large number of protein involved in copper homeostasis are pathogenic: *Klebsiella pneumoniae* NTUH-K2044, *Klebsiella pneumoniae* subsp. pneumoniae MGH 78578, *Enterobacter cloacae* subsp. cloacae ATCC 13047 and *Escherichia coli* 55989 are human pathogens; *Escherichia coli* APEC O1 is a chicken pathogen and *Escherichia coli* ATCC 8739, *Cronobacter sakazakii* ATCC BAA-894 and *Cronobacter turicensis* TAX413502 may be opportunistic organisms. Although these organisms are well characterized, no relevant information about their biology or their lifestyles explained why these organisms present the largest repertoire of copper tolerance proteins. On the other hand, 5% of the organisms (all of them intracellular parasites) apparently lack copper homeostasis proteins. In the remaining organisms, the ensemble consolidated in four clusters: PcoC-CueO-YebZ-CutF-CusF, PcoE-PcoD, PcoA-PcoB and CusC-CusA-CusB-CopA, that pointed the most frequent strategies to address the necessary copper homeostasis. In this context, it is remarkable that the observed clusters were not fully consistent with evidence obtained from transcriptional co-regulation which has been fundamental for systems designation. In general, clusters distributed with phylogenetic consistency at the family level, suggesting inheritance as the main mechanism for gene transfer. However, in some organisms harboring the full copper homeostasis repertoire, genes were organized as islands in plasmids and flanked by mobile elements, enabling them with the potential to be horizontally transferred (Additional file 2). Double optimization of the presence/absence profile exposed a tight organization of the seed proteins into nine different repertoires revealing the diversity of copper homeostasis in gamma proteobacteria.

Redundancy is a common approach to improve the reliability and availability of a system. Adding redundancy increases the cost and complexity of a system design but if the cost of failure is high enough, redundancy may be an attractive option. In the case of copper metabolism in gamma proteobacteria, maintaining all 14 proteins may result metabolically expensive for bacteria and the cost-benefit relationship would be positive only when the organism has to survive in a highly toxic environment with frequent oscillations in oxygen concentration. However, most microorganisms do not regularly deal with this kind of environment and have thus assembled different combinations of the three basic functions: transport across the plasma membrane, periplasmic chaperoning, and transport across the outer membrane.

When the distribution is observed through the whole ensemble, it is possible to identify two functions as predominant: an inner membrane pump to extrude copper from the cytoplasm to the periplasm (CopA) and an external membrane pump to export copper to the extracellular matrix (CusC). CopA performs the essential role of cytoplasmic Cu^+^ efflux across the plasma membrane [25-27]. This protein belongs to the P-ATPases superfamily which is widely distributed across all kingdoms and it has been suggested that in prokaryotes and some unicellular eukaryotes its primary function may be to protect cells from extreme environmental conditions, indicative of a vital and perhaps ancestral function [28,29]. There is limited information regarding the evolutionary history of CopA although the potential role that lateral gene transfer might have played in the evolution of P_IB_-type ATPases, in contrast to other genes involved in survival in metal-stressed environments, has been addressed [30].

The RND efflux pump superfamily is present in all kingdoms and a major role in the intrinsic and acquired tolerance to antibiotics and other toxic compounds including metal ions [31,32]. The Cus system belongs to the RND superfamily and shares their tripartite composition: a substrate-binding inner membrane transporter (CusA), a periplasmic connecting protein (CusB) and an outer membrane-anchored channel (CusC) [33,34] CusC was the second more frequently found copper tolerance protein in gamma proteobacteria, however 52 organisms harboring CusC lacked CusAB.

An appealing feature was the identification of a hybrid cluster composed of two outer membrane proteins, one inner membrane protein, and two periplasmic proteins (PcoC-CueO-YebZ-CutF-CusF) common to most Enterobacteria but absent from any other family. YebZ do not belong to current copper homeostasis systems but has been identified as a PcoD homolog [7], it is important to notice that *pcoD* is locate on plasmids in the 33% of the organism and flanked by transposases, while *yebZ* is always chromosomal. In this regard, not only the presence of PcoD was limited but also that of PcoE and CueP. We were unable to identify other PcoE or CueP homologs indicating that they might have been recruited in recent and particular adaptation events. CueP has been described as part of the Cue system in *Salmonella* based on its regulation by CueR and was suggested to compensate the lack of the Cus system under anaerobic conditions [5]. However, we identified the coexistence of CueP with CusABC only in *Pectobacterium*, *Shewanella*, *Citrobacter* and *Ferrimonas*.

Among the periplasmic copper carriers, PcoA and CueO belong to the multicopper oxidase family, sharing an ancestral fold which has evolved by duplication and specialization events. The fold has been linked to three different functions in bacteria: oxidoreductase, copper chaperone, or cell division factor. PcoA and CueO perform a particular case of oxidation activity of cuprous ions [35]. CueO is mainly found in Enterobacteria whereas PcoA is characteristic of Pseudomonadales and Xanthomonadales, being the presence of both proteins mutually exclusive.

### Evolution of copper homeostasis in gamma proteobacteria

Diverse biochemical, genetic and crystallographic studies have been performed to characterize the different proteins involved in copper tolerance in gamma proteobacteria [11,13,15,25,33,36]. In this paper we analyzed the current copper homeostasis model, where systems are the evolutionary and functional unit, from a phylogenomic perspective.

It can be observed from our results that copper homeostatic systems do not behave as evolutionary units but particular species assemble different combinations of basic functions. To explain this behavior we propose that the process by which bacteria handle copper can be compared to a metabolic pathway since organisms avoid free copper ions within the cell by developing copper translocation routes based in precise sequences of specific protein-protein interactions [16-18]. There are currently different models aimed at explaining the evolution of metabolic pathways. The patchwork hypothesis postulates that duplication of genes encoding primitive and promiscuous enzymes (capable of catalyzing various reactions) allows each descendant enzyme to specialize in one of the ancestral reactions, this model considers the chemical mechanism as dominant [37]. Alternatively, the retrograde hypothesis suggests that, in the case where a substrate tends to be depleted, gene duplication can provide an enzyme capable of supplying the exhausted substrate, giving rise to homologous enzymes catalyzing consecutive reactions with the implicit assumption that substrate specificity is dominant [38].

Assuming that the selectable phenotype would be the control of copper concentration in the cellular space in response to its availability, the fitness value would rely first on the ability of proteins for copper binding (a trait previously and independently acquired) and then on the affinity and specificity of protein-protein interactions. Following these considerations, we propose two alternative hypotheses for the evolution of copper homeostasis in gamma proteobacteria: 1) Function is dominant. 2) Protein-protein interaction is dominant. In the first case and assuming each protein fulfills a specific function among the three known for copper homeostasis proteins in bacteria, its occurrence in a repertoire will be determined by functional complementation and not by stringent protein-protein interactions. In this case, we do not expect specific pairs to be frequently found but functional patterns to occur. Considering the second possibility, two copper-binding proteins with low capacity for general protein-protein interactions will develop stronger affinity and specificity for the interaction between them until the pair is fixed. In consequence, they will be expected to coexist in different genomes and probably to be co-regulated.

To analyze these options, we will focus on two well-characterized protein combinations, the PcoA/PcoC pair and the CusABCF group. The interaction between PcoA and PcoC and its role in the oxidation of Cu(I) to the less toxic Cu(II) has been previously demonstrated [39]. This evidence would suggest that the presence of both proteins might correlate. However, our results demonstrate that in those organisms where PcoC was identified its presence correlated more strongly with CueO than with PcoA, being the latter protein frequently found by itself. Furthermore, only in organisms with high number of copper homeostasis proteins *pcoA* and *pcoC* are adjacent (along with the rest of the Pco system) whereas the most frequent arrangements were the co-localization of *pcoA* with *pcoB* and of *pcoC* with *yebZ*, a homolog of PcoD, supporting the previously suggested interaction between these two last proteins to form a functional unit replacing PcoC-PcoD [7]. A revealing piece of evidence suggesting novel interactions arises from the high frequency of co-localization of *pcoA* and *pcoB* including the detection of fused PcoA and PcoB in five *Legionella* species.

The second protein combination is the CusA-CusB-CusC group that in *E. coli* assembles as a tripartite efflux complex with the ratio CusA_3_-CusB_6_-CusC_3_ (Figure 2). Each one of the proteins has been demonstrated by different methods to independently bind copper [12]. Initial experiments using lysine-lysine cross-linking coupled with LC-MS/MS suggested the close interaction of CusA and CusB [40]; interaction further corroborated by the 2.9 Å crystallographic structure of a CusA-CusB co-crystal [33]. Putative interactions between CusC and CusA/CusB have been proposed on the basis of molecular dynamics yielding a trans-envelope structure resembling the architectures of the OprM and TolC channels [41]. The specific interaction of CusB with CusF, a small periplasmic protein with a putative role as a methallochaperone, as metal transfer partners has been demonstrated by isothermal titration calorimetry, XSAFS and NMR [42].

Once again, this evidence leads to the expectation for these four proteins to coexist and even to be co-localized in the genome. The CusABCF group was found in 21 families of 12 different orders but with evidence of co-localization only in Enterobacteria (*Escherichia coli*, *Citrobacter*, *Cronobacter*, *Shigella*, *Klebsiella*, *Edwardsiella* and *Enterobacter*) and in one other species (*Shewanella putrefaciens* CN-32 and ANA-3). The most frequent presence patterns for these proteins were CusC by itself followed by CusA-CusB-CusC. CusB was found by itself in only one organism and it was more frequent to find CusA and CusC without CusB, suggesting a less stringent role of CusB as an accessory for the formation of the CusA-CusC structure. CusF was identified in only five families and in 62% of them it co-localized with *cusABC*. However, the fact that in 22 organisms CusB and CusF were fused in a single gene do not compare with the role of CusF as a soluble carrier, a role that certainly deserves to be revised. In *E. coli* APEC 01 we identified a CusABC paralog, named SilABC which is plasmid borne and adjacent to PcoAB, with an apparent role in silver extrusion suggesting evolution by duplication and functional equivalence but metal-binding specialization.

These analyses were performed with the aim to elucidate between two hypotheses for the concurrent evolution of well characterized interacting protein sets in copper homeostasis: function dominance or protein-protein interaction dominance, The high presence correlation of CusABC support protein-protein interaction as the selection trait for the assembly with two caveats: CusC may still be functional in the absence of CusAB (as happens in other RND groups, [43]). This idea is consistent with the fact that in a number of cases *cusC* was found to lie adjacent to genes encoding for RND complexes with other proposed specificities. Additionally it would be interesting to determine if the minimal set of an inner membrane protein such CopA and a single outer membrane protein such as CusC are sufficient for copper tolerance acquisition.

In contrast, the low presence correlation between PcoA/PcoC compared to the higher and unexpected correlation of PcoC with CueO may lead to observation that CueO functionally replaces PcoA on the interaction with PcoC. However, CueO and PcoA belong to the MCO structural family and, in spite of sharing low identity at the sequence level, their three dimensional structure is highly preserved as happens with the rest of the family members [44]. In both cases evidence support the protein-protein interaction hypothesis as the basic mechanisms for the evolution of the copper homeostasis systems supporting our theoretical treatment as metabolic networks [45].

## Conclusions

Our results suggest complex evolutionary dynamics and still unexplored interactions among different proteins to achieve copper homeostasis in gamma proteobacteria, challenging some of the molecular transport mechanism proposed for these systems.

## Methods

### Gamma proteobacterial genomes

To carry out this analysis we analyzed 268 proteobacterial genomes available from the KEGG database (Release 56.0, October 1, 2010) [46,47] (Aditional file 1).

### Protein sequences used as seeds for ortholog detection

CopA from *Escherichia coli* K-12 MG1655 [KEGG:eco:b0484]; CueO from *Escherichia coli* O1:K1:H7 (APEC) [KEGG:ecv:APECO1_1862]; CueP from *Salmonella enterica* subsp. enterica serovar typhimurium LT2 [KEGG:stm:STM3650]; CusA from *Escherichia coli* K-12 MG1655 [KEGG:eco:b0575]; CusB from *Escherichia coli* K-12 MG1655 [KEGG:eco:b0574]; CusC from *Escherichia coli* K-12 MG1655 [KEGG:eco:b0572]; CusF from *Escherichia coli* K-12 MG1655 [KEGG:eco:b0573]; PcoA from *Escherichia coli* O1:K1:H7 (APEC) [KEGG:ecv:APECO1_O1R119.2]; PcoB from *Escherichia coli* O1:K1:H7 (APEC) [KEGG:ecv:APECO1_O1R119]; PcoC from *Escherichia coli* O1:K1:H7 (APEC) [KEGG:ecv:APECO1_O1R120]; PcoD from *Escherichia coli* O1:K1:H7 (APEC) [KEGG:ecv:APECO1_O1R121]; PcoE from *Escherichia coli* O1:K1:H7 (APEC) [KEGG:ecv:APECO1_O1R118]; YebZ from *Escherichia coli* O1:K1:H7 (APEC) [KEGG:ecv:APECO1_893]; CutF from *Escherichia coli* O1:K1:H7 (APEC) [KEGG:ecv:APECO1_1795].

### Bidirectional best hit orthology criterion

The bidirectional best hit (BBH) criterion is a widely used procedure for orthology assessment of a seed sequence in a target genome resulting in a group of hits, being one of them the best match [48]. This match becomes *bidirectional* when both sequences (seed and target) result to be the best hit for each other. A *bidirectional best hit* represents a very strong similarity between two genes and is considered evidence that the genes may be orthologs [48,49]. BBH criterion uses BLASTP with a cut E-value of 10^-3^ and minimal alignment coverage for query and/or subject sequence ≥ 50%. (Additional file 1).

### Phylogenetic profile construction

We constructed two different phylogenetic profiles, one at the species and the other one at the genus level. The phylogenetic profile at the species level was constructed by assigning a value of 1 when an ortholog was identified in a genome and a value of 0 when not, using species as clades [50]. The phylogenetic profile at the genus level was constructed assigning values representing the fractional abundance corresponding to the percentage of a seed protein within a given genera, in this case, clades represent all analyzed genus. To facilitate handling and data representation, values were organized in 11 discrete intervals between 0 and 1.

### Clustering

Data clustering was performed using the Hierarchical Clustering algorithm in the Multiexperiment viewer software [51,52]. For matrix optimization, we used Pearson distance as a metric for tree calculation and average linkage to indicate distances between clusters. To define clusters we use CAST tool (Clustering Affinity Search Technique) from the same software.

### Phylogenetic tree construction

We selected one representative genome form each genus following KEGG classification [46,47] and we used the taxonomic Id from NCBI databases [53,54] to build a phylogenetic tree with the Interactive Tree Of Life (iTOL) [55,56]. Dendroscope was used to manipulate the tree [57].

## Abbreviations

BBH: Bidirectional best hit; MCO: Multicopper oxidase.

## Competing interest

The authors declare that they have no competing interest.

## Authors’ contributions

GHM performed computational analyses. BV, JMA and GHM were involved in conception and interpretation of the results and drafting the manuscript. BV, JMA and GHM were involved in critically revision the manuscript for intellectual content and approved the manuscript for publication. All authors read and approved the final manuscript.

## Supplementary Material

Additional file 1 **The following additional data are available with the online version of this paper.** Additional data file 1 is a excel spreadsheet listing the 268 organisms used in this study, and a table listing all orthologs obtain by the Bidirectional Best Hit. Click here for file

Additional file 2 **The following additional data are available with the online version of this paper.** Additional data file 2 is a table listing PcoC proteins in 8 organisms harboring the full copper homeostasis repertoire, indicating location and presence of mobile elements. Click here for file
